# A Precise Error Bound for Quantum Phase Estimation

**DOI:** 10.1371/journal.pone.0019663

**Published:** 2011-05-10

**Authors:** James M. Chappell, Max A. Lohe, Lorenz von Smekal, Azhar Iqbal, Derek Abbott

**Affiliations:** 1 School of Chemistry and Physics, University of Adelaide, Adelaide, South Australia, Australia; 2 Institut für Kernphysik, Technische Universität Darmstadt, Darmstadt, Germany; 3 School of Electrical and Electronic Engineering, University of Adelaide, Adelaide, South Australia, Australia; Humboldt University, Germany

## Abstract

Quantum phase estimation is one of the key algorithms in the field of quantum computing, but up until now, only approximate expressions have been derived for the probability of error. We revisit these derivations, and find that by ensuring symmetry in the error definitions, an exact formula can be found. This new approach may also have value in solving other related problems in quantum computing, where an expected error is calculated. Expressions for two special cases of the formula are also developed, in the limit as the number of qubits in the quantum computer approaches infinity and in the limit as the extra added qubits to improve reliability goes to infinity. It is found that this formula is useful in validating computer simulations of the phase estimation procedure and in avoiding the overestimation of the number of qubits required in order to achieve a given reliability. This formula thus brings improved precision in the design of quantum computers.

## Introduction

Phase estimation is an integral part of Shor's algorithm [1] as well as many other quantum algorithms [2], designed to run on a quantum computer, and so an exact expression for the maximum probability of error is valuable, in order to efficiently achieve a predetermined accuracy. Suppose we wish to determine a phase angle 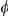 to an accuracy of 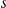 bits, which hence could be in error, with regard to the true value of 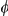, by up to 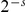, then due to the probabilistic nature of quantum computers, to achieve this we will need to add 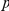 extra qubits to the quantum register in order to succeed with a probability of 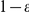. Quantum registers behave like classical registers upon measurement, returning a one or a zero from each qubit. Previously, Cleve et al. [3] determined the following upper bound:
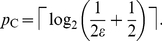
(1)


Thus the more confident we wish to be (a small 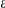), for the output to achieve a given precision 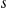, the more qubits, 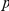, will need to be added to the quantum register. Formulas of essentially the same functional form as Eq. (1), are produced by two other authors, in [2] and [4], due to the use of similar approximations in their derivation. For example, we have 
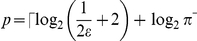
, given in [4]. As we show in the following, these approximate error formulas are unsatisfactory in that they overestimate the number of qubits required in order to achieve a given reliability.

The phase angle is defined as follows, given a unitary operator 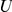, we produce the eigenvalue equation 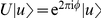, for some eigenvector 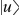, and we seek to determine the phase 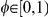 using the quantum phase estimation procedure [5]. The first stage in phase estimation produces, in the measurement register with a 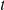 qubit basis 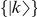, the state [2]

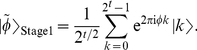
(2)


If 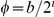 for some integer 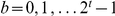, then
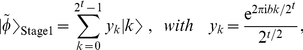
(3)is the discrete Fourier transform of the basis state 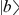, that is, the state with amplitudes 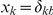. We then read off the exact phase 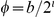 from the inverse Fourier transform as 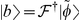.

In general however, when 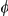 cannot be written in an exact 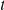 bit binary expansion, the inverse Fourier transform in the final stage of the phase estimation procedure yields a state
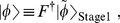
(4)from which we only obtain an estimate for 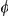. That is, the coefficients 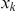 of the state 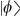 in the 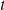 qubit basis 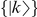 will yield probabilities which peak at the values of 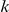 closest to 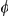.

Our goal now is to derive an upper bound which avoids the approximations used in the above formulas and hence obtain a precise result.

## Results

In order to derive an improved accuracy formula for phase estimation, we initially follow the procedure given in [3], where it is noted, that because of the limited resolution provided by the quantum register of 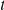 qubits, the phase 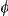 must be approximated by the fraction 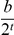, where 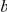 is an integer in the range 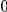 to 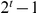 such that 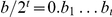 is the best 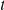 bit approximation to 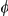, which is less than 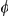. We then define
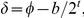
which is the difference between 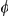 and 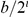 and where clearly 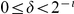. The first stage of the phase estimation procedure produces the state given by Eq. (2). Applying the inverse quantum Fourier transform to this state produces
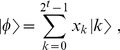
(5)where
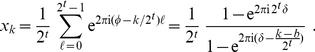
(6)


Assuming the outcome of the final measurement is 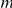, we can bound the probability of obtaining a value of 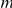 such that 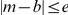, where 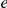 is a positive integer characterizing our desired tolerance to error, where 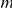 and 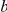 are integers such that 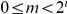 and 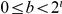. The probability of observing such an 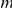 is given by
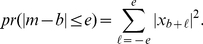
(7)


This is simply the sum of the probabilities of the states within 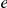 of 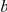, where
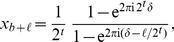
(8)which is the standard result obtained from Eq. (6), in particular see equation 5.26 in [2]. Typically at this point approximations are now made to simplify 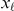, however we proceed without approximations. We have
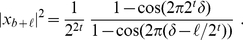
(9)


Suppose we wish to approximate 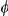 to an accuracy of 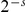, that is, we choose 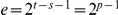, using 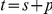, which can be compared with Eq. 5.35 in [2], and if we denote the probability of failure
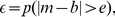
(10)then we have
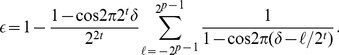
(11)


This formula assumes that for a measurement 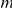, we have a successful result if we measure a state either side of 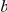 within a distance of 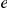, which is the conventional assumption.

This definition of error however is asymmetric because there will be unequal numbers of states summed about the phase angle 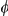 to give the probability of a successful result, because an odd number of states is being summed. We now present a definition of the error which is symmetric about 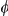.

### Modified definition of error

Given an actual angle 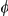 that we are seeking to approximate in the phase estimation procedure, a measurement is called successful if it lies within a certain tolerance 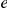 of the true value 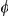. That is, for a measurement of state 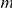 out of a possible 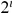 states, the probability of failure will be
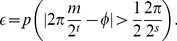
(12)


**Figure 1:**
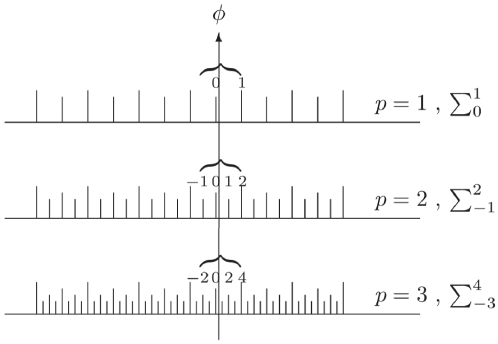
Defining the limits of summation for the phase estimation error. For the cases 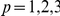, we show the measurements which are accepted as lying within the required distance of 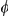, shown by the vertical arrow, which define the limits of summation used in Eq. (13).

Thus we consider the angle to be successfully measured accurate to 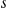 bits, if the estimated 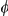 lies in the range 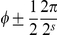. Considering our previous definition Eq. (10), due to the fact that 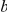 is defined to be always less than 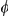, then compared to the previous definition of 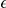, we lose the outermost state at the lower end of the summation in Eq. (11) as shown in Fig. (1). For example for 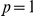, the upper bracket in Fig. (1) (representing the error bound) can only cover two states instead of three, and so the sum in Eq. (11) will now sum from 0 to 1, instead of 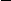
1 to 1, for this case.

### An optimal bound

Based on this new definition then for all cases we need to add 1 to the lower end of the summation giving
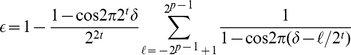
(13)and if we define 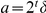 and rearrange the cosine term in the summation we find
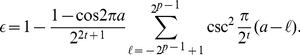
(14)


Next, we demonstrate that the right hand side of Eq. (14) takes its maximum value at 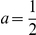. Since we know 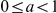, and since we expect the maximum value of 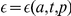 to lie about midway between the two nearest states to generate the largest error, that is at 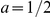, we will substitute 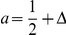, where 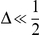. To maximize 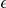 we need to minimize
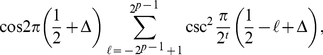
(15)as a function of 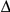. Expanding to quadratic order with a Taylor series, we seek to minimize
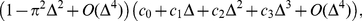
(16)where 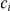 are the coefficients of the Taylor expansion of cosecant^2^ in 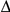. We find by the odd symmetry of the cotangent about 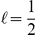 that
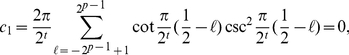
(17)and so we just need to minimize
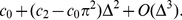
(18)


Differentiating, we see we have an extremum at 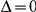, and therefore 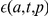 has a maximum at 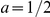.

Substituting 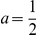 we obtain
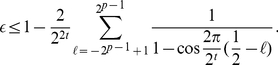
(19)


We note that the summation is symmetrical about 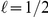, and substituting 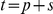, we obtain for our final result
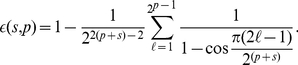
(20)


That is, given a desired accuracy of 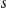 bits, then if we add 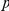 more bits, we have a probability of success given by 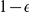, of obtaining a measurement to at least 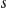 bits of accuracy. Thus we have succeeded in deriving a best possible bound for the failure rate 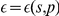.

### Special Cases

Numerical calculations show that 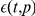 quickly approaches its asymptotic value as 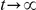, and this limit gives a fairly accurate upper bound for 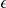, for 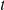 greater than about 10 qubits. Using 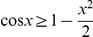 which is valid for all 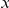, and is accurate for 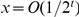 as 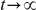,
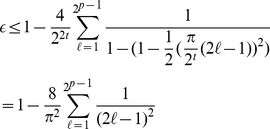
(21)


An exact form for this can be found in terms of the trigamma function, being a special case of the polygamma function as shown in Abramowitz and Stegun [6], Eq. 6.4.5:
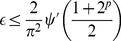
(22)where 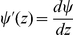 is the trigamma function, 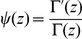 is the digamma function, and 
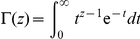
 is the standard gamma function.

Now considering the 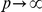 limit, which also includes the 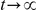 limit because 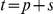, we can find an asymptotic form in the limit of large 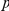 also from [6], Eq. 6.4.12, namely
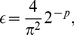
(23)which shows that the error rate drops off exponentially with 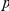 extra qubits. The formula Eq. (23) can be re-arranged to give
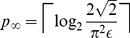
(24)which can be compared with the previous approximate formula shown in Eq. (1).

We have checked the new error formula through simulations, by running the phase estimation algorithm on a 2-dimensional rotation matrix, and undertaking a numerical search for the rotation angle that maximizes the error 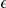, which has confirmed Eq. (20) to six decimal places.

## Discussion

An exact formula is derived for the probability of error in the quantum phase estimation procedure, as shown in Eq. (20). That is, to calculate 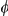 accurate to a required 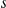 bits with a given probability of success 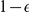 we add 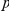 extra qubits, where 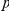 is given by Eq. (20). If we have a large number of qubits then we can use Eq. (22) valid at the 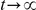 limit. In the 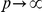 limit the asymptote is found as a simple exponential form Eq. (23).

The exact formula avoids overestimating the number of qubits actually required in order to achieve a given reliability for phase estimation and we have also found this formula to be useful in confirming the operation of classical simulators of the phase estimation procedure.

## References

[1] Shor PW (1997). Polynomial-time algorithms for prime factorization and discrete
logarithms on a quantum computer.. SIAM, J Comp.

[2] Nielsen MA, Chuang IL (2002). Quantum Computation and Quantum Information..

[3] Cleve R, Ekert A (1998). Quantum algorithms revisited.. Proc R Soc London A.

[4] Imre S, Balazs F (2002). A tight bound for probability of error for quantum counting based
multiuser detection.. Proc ISIT.

[5] Mosca M (1999). Quantum Computer Algorithms..

[6] Abramowitz M, Stegun IA (1964). Handbook of Mathematical Functions with Formulas, Graphs, and
Mathematical Tables..

